# Vitamin D3 attenuates doxorubicin-induced senescence of human aortic endothelial cells by upregulation of IL-10 via the pAMPKα/Sirt1/Foxo3a signaling pathway

**DOI:** 10.1371/journal.pone.0252816

**Published:** 2021-06-08

**Authors:** Lei Chen, Rachel Holder, Charles Porter, Zubair Shah

**Affiliations:** Department of Cardiovascular Medicine, University of Kansas Medical Center, Kansas City, Kansas, United States of America; Max Delbruck Centrum fur Molekulare Medizin Berlin Buch, GERMANY

## Abstract

The toxicity of doxorubicin to the cardiovascular system often limits its benefits and widespread use as chemotherapy. The mechanisms involved in doxorubicin-induced cardiovascular damage and possible protective interventions are not well-explored. Using human aortic endothelial cells, we show vitamin D3 strongly attenuates doxorubicin-induced senescence and cell cycle arrest. We further show the protective effects of vitamin D3 are mediated by the upregulation of IL-10 and FOXO3a expression through fine modulation of pAMPKα/SIRT1/FOXO3a complex activity. These results have great significance in finding a target for mitigating doxorubicin-induced cardiovascular toxicity.

## Introduction

Doxorubicin (DOX) continues to be a frontline, broad-spectrum, anticancer agent. Serious cardiotoxic effects, ultimately leading to heart failure, have limited the widespread use of DOX in cancer patients [1]. There has been recent evidence indicating DOX induces endothelial cell damage and death [2–4]. The endothelial lining of coronary blood vessels plays a vital role in maintaining cardiomyocyte function and health by providing a protective barrier, promoting the delivery of nourishment, and releasing paracrine factors [5]. Thus, any damage to the endothelium can multiply the cardiotoxic potential of DOX, leading to more severe cardiomyopathy. Furthermore, DOX-induced endothelial dysfunction can enhance vascular damage via known risk factors, such as the inflammatory milieu, hyperlipidemia, hypertension, and diabetes mellitus [6]. This can lead to a progressive decline in cardiomyocyte health and contribute to chronic cardiomyopathy. The potential of DOX to compromise vascular health has led to a recent paradigm shift, making the endothelium a novel upstream target for the prevention of DOX-induced cardiomyopathy.

Numerous studies have indicated DOX causes cell damage via oxidative stress and subsequent inflammation [3, 7–9]. Therefore, DOX-induced oxidative stress and inflammation leading to endothelial cell senescence represent novel pathways to explore for the prevention of DOX-induced cardiomyopathy. Interleukin 10 (IL-10) has emerged as a possible target within these pathways. IL-10 is an anti-inflammatory cytokine that works by downregulating the transcription of pro-inflammatory cytokines [10]. Studies have shown the lack of IL-10 leads to increased DOX-induced cardiotoxicity in murine models [11]. Therefore, overexpression or upregulation of IL-10 may potentially mitigate DOX-associated cardiovascular toxicity.

Additionally, vitamin D3 (VitD3) has been implicated as a mediator of cardiovascular hemostasis, and its deficiency has been associated with cardiovascular dysfunction [12–14]. Endothelial cells synthesize VitD3 and express vitamin D receptors (VDR), indicating a possible autocrine mechanism for regulation of endothelial functions [12]. Although the relationship among VitD3, endothelium, and cardiovascular disease is well established, little is known about the effects of VitD3 on endothelial cells in response to DOX-induced oxidative stress and senescence. A multitude of studies indicate VitD3 is a safe and effective supplement for cancer patients and has anti-tumor effects via inhibition of cancer cell proliferation and invasiveness, induction of differentiation, and apoptosis [15–17]. Furthermore, VitD3 has been safely used with chemotherapeutic agents, including DOX, in cancer patients with evidence that it may sensitize various cancer cells to chemotherapy [18–20]. The known selective, pro-senescence effects of VitD3 on cancer cells and protective effects against oxidative stress and senescence in the cardiovascular system make it an excellent agent to be studied in the prevention of DOX-induced cardiovascular toxicity.

In this study, we investigate the potential of VitD3 in the prevention of DOX-induced senescence in human aortic endothelial cells (HAECs). Our results highlight that VitD3 has protective effects against DOX-mediated endothelial damage through the upregulation of IL-10 expression via the AMPKα/SIRT1/FOXO3a signaling pathway.

## Materials and methods

### Antibodies and reagents

Multiple reagents and kits were used throughout this study. A detailed description of these reagents is available in S1 Table.

### The primary human aortic endothelial cell culture and treatment

HAECs were purchased from Cell Application Inc (304k-05a). These cells were isolated from both the ascending and descending aortas of humans. Cell culture flasks were first coated with 2% gelatin for 30 minutes. The HAECs were seeded into the gelatin-coated flasks and cultured in Human Endothelial Cell Growth Medium (ECGM) media. The cells were considered ready to use for experiments once they reached 80% to 90% confluence. Transient transfections were performed using Invitrogen™ Lipofectamine™ 3000 reagent following the manufacture’s recommendations.

### IL-10 activity neutralization

IL-10 activity neutralization was performed with the administration of additional IL-10 antibody to prevent IL-10 from binding to its receptor. According to published literature [21, 22], the anti-human IL-10 mAb 9D7 was administrated into the culture media at the final concentration of 10 μg/ml, to neutralize IL-10 activity. An equal amount of normal rat IgG1 (Santa Cruz Biotechnology) was added as a control to other groups. After combining with VitD3 and DOX treatment, total RNA of the HAECs was collected and qPCR was performed to quantify the effect of neutralizing IL-10 activity on senescence-related genes.

### Lentiviral shRNAs

shRNA lentivirus particles were purchased from MilliporeSigma. The shRNA target sequences are listed below: Sirt1 target Sequence:

5’-ccggcaggtcaagggatggtatttactcgagtaaataccatcccttgacctgtttttg-3’;

Foxo3a shRNA lentivirus particle sequence:

5’-ccggcatgttcaatgggagcttggactcgagtccaagctcccattgaacatgtttttg-3’.

The non-target shRNA control transduction particles:

5’-ccgggcgcgatagcgctaataatttctcgagaaattattagcgctatcgcgcttttt-3’. Following the protocol, HAECs were infected by the lentivirus particles for 48 hours before selection by 2 μg/ml puromycin (MilliporeSigma, P8833).

### Gene expression analysis by quantitative real-time RT-PCR

Total RNA was collected from HAECs by TRIzol reagent (Invitrogen, 15596018). Next, total RNA was reversed transcribed into cDNA by the first-strand cDNA synthesis system (Bio-Rad, 170–8841) according to the manufacture’s protocol. The expression of genes related to senescence were examined by quantitative real-time PCR using the ViiA™ 7 Real-Time PCR System (Applied Biosystems). The housekeeping gene *18S* was used as an internal control.

Real-time PCR was performed using EvaGreen qPCR Master Mix (MidSci, BEQPCR-LR), cDNA template, forward primers, and reverse primers (S2 Table). The comparative Ct method was used to calculate the relative quantification of gene expression levels. The relative quantitative values of the targets were normalized to the *18S* control and compared to a calibrator. Results were expressed as 2^−ΔΔ*Ct*^ (i.e.-fold difference).

### Immunoblot

Cell extracts were lysed in PierceTM IP lysis buffer (Thermo Fisher Scientific, 87788) with a supplement of protease inhibitors (Roche Diagnostics, 04693124001). Proteins were harvested by centrifugation and concentrated using the Pierce BCA Protein Assay kit (Thermo Fisher Scientific, 23224). The concentrated, normalized protein samples were prepared in SDS-polyacrylamide gel electrophoresis (SDS-PAGE) loading buffer, run on 10% SDS-PAGE gel, and transferred onto PVDF membrane (LI-COR Biosciences, 926–32098) using the Wet/Tank Blotting Systems (BioRad, 1703930). After blocking with Intercept® Blocking Buffer (LI-COR Biosciences, 927–70001), the membrane was exposed to a blocking buffer containing primary antibodies. After washing and secondary antibody (LI-COR Biosciences, 925–68072, and 925–32213) labeling, the positive protein bands were detected and visualized using an Odyssey scanner (LI-COR Biosciences). Densitometry analysis was achieved using the Image Studio software supplied by LI-COR Bioscience.

### Immunofluorescence

The HAECs were plated on glass-bottom dishes precoated with gelatin. After pretreating with their designated DOX and/or VitD3 dose, the HAECs were rinsed with PBS, fixed with 4% formaldehyde, and permeabilized with 0.3% Triton X-100 using the BD cytofix/cytoperm fixation/permeabilization kit (BD biosciences, 554714). After rinsing with PBS twice and being blocked with 10% normal horse serum (Vector Laboratory, S-2000) in PBS for 1 hour, the HAECs were incubated with primary antibodies (diluted with 10% horse serum in PBS) overnight at 4°C. This was followed by a wash and exposure to a 10% horse serum in PBS diluted fluorescently labeled secondary antibodies (Vector Laboratory, DI-1088, DI-1094 or/and DI-2549) for one hour in the dark at room temperature. After completely washing with PBS and mounting with VECTASHIELD® Antifade Mounting Medium with DAPI (Vector Laboratory, H-1200), the fluorescence of HAECs was examined with a Leica laser point scanning confocal microscope (Leica TCS SPE).

### Cell amplification

HAECs were trypsinized and cultured at low density (10,000 cells/ml) in gelatin-coated, 6-well dishes. The HAECs were allowed 24 hours in ECGM-V2 media for attachment and growth. The cells were then treated with VitD3 and/or DOX at varying doses. Cells were harvested at specific time points post-treatment (12, 24, 36, and 48 hours), dissociated with 0.05% Trypsin/EDTA, and counted under the Invitrogen™ Countess™ II Automated Cell Counter (Invitrogen, AMQAX1000). The results were quantified after data was obtained from three independent experiments performed at different times.

### Flow cytometry for cell cycle assay using PI

Cell cycle analysis of HAECs was performed with flow cytometry by BD PI/RNase staining buffer (BD, BDB550825). After treatment with VitD3 and/or DOX, the HAECs were harvested, dissociated with trypsin, then washed with cold PBS. The collected cells were then fixed with 1% paraformaldehyde for 15 min at 4°C. Next, they were stained with propidium iodide (PI) solution containing 50 mg/mL RNase A, and then, they were analyzed using the BD Biosciences Coulter machine (BD^TM^ LSR II). Data obtained was analyzed and presented using FlowJo software (FlowJo LLC).

### Chromatin immunoprecipitation

The VDR binding region in the *FOXO3a* promoter and FOXO3a binding motif in the *IL-10* promoter region were first analyzed through JASPAR (http://jaspardev.genereg.net/). For the Chromatin Immunoprecipitation (CHIP) assay, cells were subjected to VitD3 treatment and then cross-linked with 1% formaldehyde for 15 mins at room temperature. The crosslinking was stopped with the addition of 125 nM glycine. Cell lysates were sonicated to generate DNA fragments with an average size of smaller than 1,000bp fragments and were immunoprecipitated with VDR specific antibody (to *FOXO3a* genomic fragments) or FOXO3a specific antibody (to *IL-10* genomic fragments). Bound DNA fragments were eluted and then amplified by PCR with specific primers (S2 Table).

### Plasmid construction and luciferase reporter assay

For the reporter assay of FOXO3a, the *FOXO3a* genomic fragment containing the wild-type VDR binding sequence (chr6+:108559555–108561092) was amplified from a commercial FOXO3a reporter vector pEZX-Foxo3a (Genecopoeia, HPRM43701) using Phusion High-Fidelity DNA Polymerase (NEB, M0530) and subcloned into the pGL3-basic vector (Promega, catalog No. E1751), named as Foxo3a-wt. The site-directed mutagenesis of the VDR binding region was performed following the Q5 Site-Directed Mutagenesis Kit protocol. Briefly, forward and reverse primers (S2 Table) were designed with the NEBase Changer (https://nebasechanger.neb.com/). The PCR was performed with Q5 High-Fidelity DNA Polymerase, two primers, and dNTP. The plasmid Foxo3a-wt was used as a temperate. After using kinase, ligase, and then digestion with DpnI enzyme to remove the temperate plasmid Foxo3a-wt, transfection was accomplished into the competent cells. The mutated construction was collected and named as Foxo3a-mut.

IL-10 reporter plasmid construction was performed with a similar procedure. Briefly, the wild-type *IL-10* promoter fragment (chr1-:206773864–206772471) from the commercial plasmids pEZX-IL10 (Genecopoeia, HPRM30536) was subcloned into the pGL3-basic vector and named as IL-10-wt. After analyzation with JASPAR, the deletion related primers (S2 Table) were designed according to the *IL-10* promoter region. Site-directed fragment deletions were generated according to the protocol of Q5 Site-Directed Mutagenesis Kit, named as pGL3-IL-10-Del1 and pGL3-IL-10-Del2.

The luciferase reporter assay was performed following the manufacture’s protocol. Briefly, the reporter vectors were first transfected into HAECs by Lipofectamine^TM^ 3000 (Invitrogen, L3000001). After 48 hours of VitD3 treatment, the luciferase activity was determined using a Dual-Luciferase Assay System (Promega, E2920). Transfection efficiency was normalized to Renilla luciferase activity co-transfected with pRL-TK vector (Promega, E2241).

### IL-10 ELISA assay

IL-10 concentration in culture media was quantified by Invitrogen human IL-10 Elisa Kit following the manufacturer instructions. In summary, the IL-10 diluted standard and culture media were added to the ELISA wells and incubated at room temperature for 2.5 hours. After four washes with washing buffer, 100 μL of Biotinylated antibody was added, and the wells were incubated for 1 hour. The wells were then washed, and 100μL of Streptavidin HRP Reagent was added to each well and incubated at room temperature for 45 mins. Once the 45 minutes were finished, the wells were washed four more times. Then, 100μL of TMB Substrate was added to the wells. The plates were then kept in the dark at room temperature for 30 mins. The reaction was stopped by adding 50μL of Stop Solution. The absorbance was measure and results were calculated.

### Statistical analysis

Statistical comparisons were performed with one-way ANOVA, or student’s *t*-tests using GraphPad Prism 8.0 software. Data were reported as mean ± standard error of mean (SEM).

## Results

### DOX reduces IL-10 levels and induces senescence in HAECs

**Fig 1:**
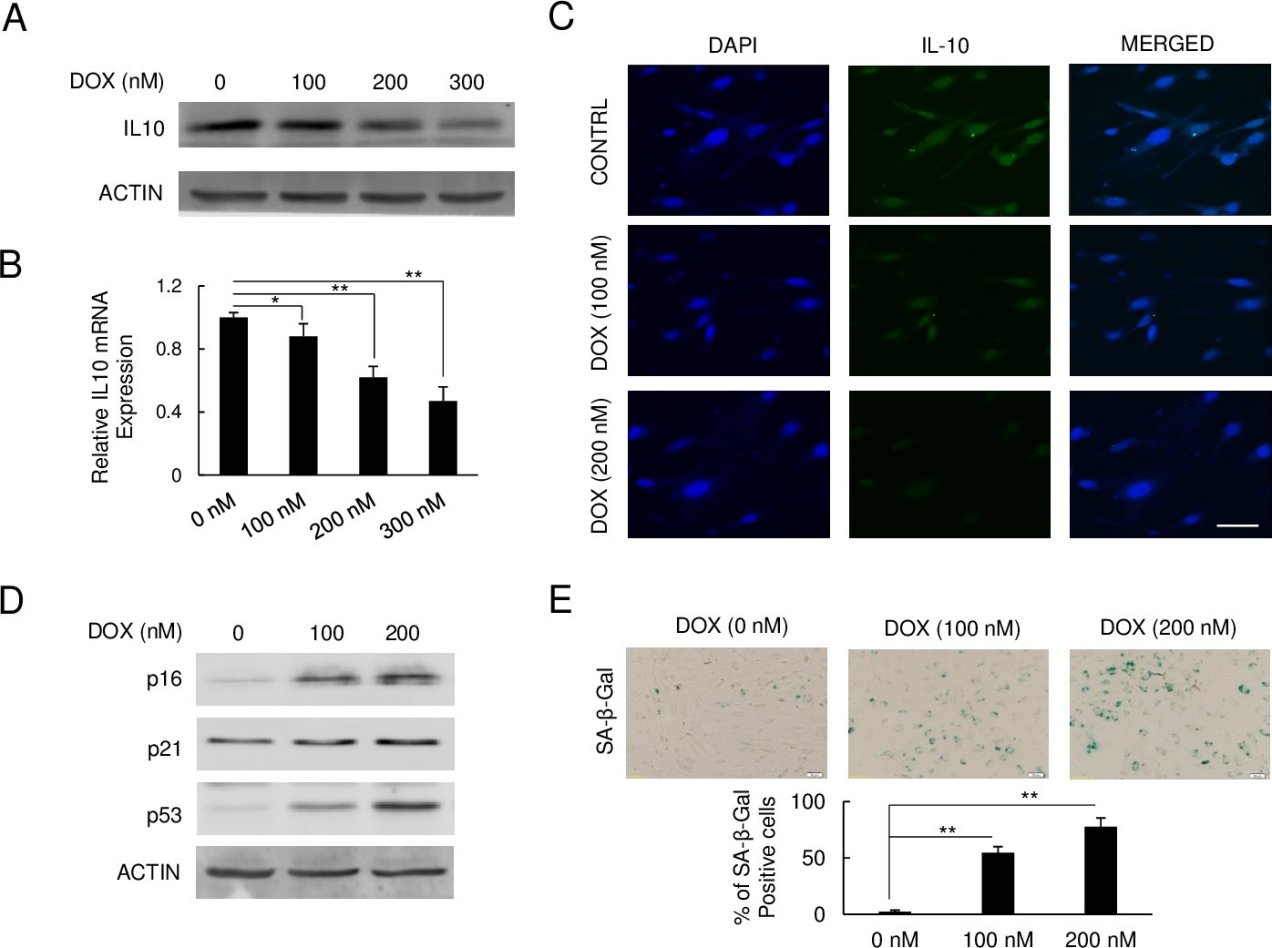
IL-10 levels decline in DOX-induced senescent HAECs. (A) Immunoblot of IL-10 after DOX treatment. (B) Relative *IL-10* mRNA expression measured by qPCR (*p < 0.05, **p < 0.01, n = 3). (C) Fluorescence staining of IL-10 on HAEC cells. IL-10 was stained with FITC-labeled and DAPI was performed for nucleus staining. Scar bar, 10 μm. (D) Immunoblot of senescence markers, p16, p21, and p53 expression levels on DOX treatment. (E) SA-β-Gal staining of senescent HAECs after DOX treatment. Senescent cells were stained with blue color under IPTG reaction (**p < 0.01, n = 5). Scar bar, 20 μm.

IL-10 activity was analyzed in DOX-treated HAECs by protein, gene, and cell-level changes through immunoblot, qPCR, and immunofluorescence staining respectively. After 48 hours of DOX treatment at 100 nM, 200 nM, and 300 nM respectively, the immunoblot analysis revealed IL-10 protein levels were strongly suppressed by DOX in a dose-dependent fashion (Fig 1A). Using quantitative real-time PCR, we further examined the mRNA expression levels of *IL-10* in HAECs exposed to DOX. As detailed in Fig 1B, the relative gene expression levels of *IL-10* were reduced by 0.88, 0.62, and 0.47-fold with 100 nM, 200 nM, and 300 nM of DOX treatment respectively compared to the control. The immunofluorescent assay revealed a decreased IL-10 fluorescence signal, which mimics variations of IL-10 mRNA quantified with qPCR in DOX treated HAECs compared to the control, thus further confirming the above findings (Fig 1C). In conclusion, DOX treatment both induced severe senescence in HAECs and suppressed IL-10 expression.

We further assessed senescence-specific markers in DOX-treated HAECs through immunoblot. As observed in Fig 1D, the p16, p21, and p53 protein levels were induced in DOX treated HAECs in a dose-response manner compared to the control. To confirm our findings and track senescence, the SA-β-gal assay was performed on DOX-treated HAECs. As shown in Fig 1E, SA-β-gal staining revealed a parallel increase in senescence of HAECs exposed to DOX in a dose-dependent fashion. The increased senescence was especially prominent for cells treated with 200 nM of DOX as compared to the control. Together, both the SA-β-gal staining and immunoblot data indicate DOX strongly induces senescence of HAECs in a dose-response fashion.

### VitD3 attenuates DOX-induced senescence and suppression of HAECs multiplication

**Fig 2:**
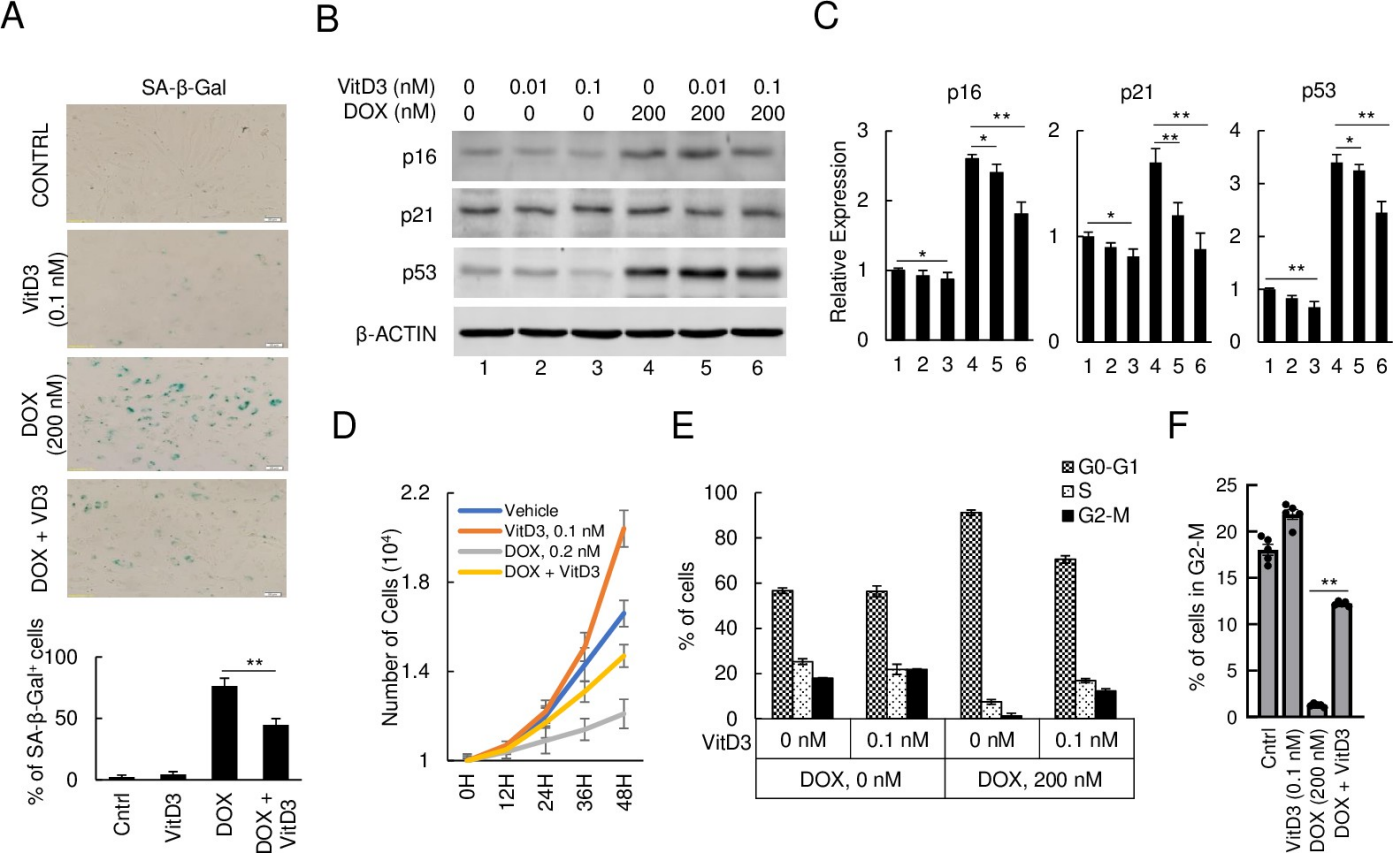
VitD3 attenuates DOX-induced senescence and cell amplification of HAECs. (A) SA-β-Gal staining of senescent HAECs after DOX or/and VitD3 treatment. Scar bar, 20 μm, (**p < 0.01, n = 3). (B) Immunoblot of p16, p21, and p53 on DOX and VitD3 cotreated HAECs. (C) Quantitative representation of the relative expression levels to the immunoblot (*p < 0.05, **p < 0.01, n = 3). (D) Cell amplification after DOX and/or VitD3 treatment and quantified the number of cells. (E) Cell cycle analysis by flow cytometry after DOX and/or VitD3 treatment. (F) Percent of cells on the G2-M stage during cell cycle analysis (**p < 0.01, n = 5).

To investigate the potential protective effect of VitD3 against DOX-induced senescence, we combined VitD3 and DOX treatment, with senescence-related gene expression, cell cycle, and cell amplification analysis. SA-β-gal staining revealed the level of senescence was markedly upregulated in the 200 nM DOX treatment group and significantly suppressed in the VitD3-DOX cotreatment group (Fig 2A). Immunoblot analysis and a corresponding densitometric assay of cellular lysates cotreated with DOX and VitD3 revealed that the p16, p21, and p53 expression levels were significantly reduced compared to the DOX-only group (Fig 2B and 2C). Both SA-β-gal staining and changes in senescence markers p16, p21, and p53 reveal VitD3 reduced the senescence in DOX-treated HAECs.

To investigate the effects of DOX and VitD3 on HAECs amplification, the cell numbers of DOX and/or VitD3 treatment groups were quantified every 12 hours. As seen in Fig 2D, VitD3 slightly promoted cell amplification when compared to the non-treated control. In contrast, DOX strongly suppressed cellular amplification starting at 12 hours and continued through the end of the timed counts compared to the control and VitD3 treated group. This DOX-induced suppression of HAECs was markedly attenuated by cotreatment with VitD3. To further investigate cell amplification, we performed flow cytometry with PI/RNase staining for cell cycle analysis at 48 hours. The G2/M phase of the cell cycle of the VitD3 only group was observed to be induced when compared to the control (Fig 2F). Treatment with 200 nM of DOX strongly suppressed cell division and kept most of the cells on the G0 phase (91.16% of DOX treated group compared to the 56.68% of control). Cotreatment with VitD3 shifted the cell cycle from DOX-induced resting phase to interphase and cell division phase (12.22% in G2/M vs. 1.26% in DOX only group) (Fig 2E and 2F). These data indicate that VitD3 neutralizes DOX-induced senescence and its effects on cell multiplication in HAECs.

### VitD3 has a protective effect on DOX-induced senescence of HAECs primarily via upregulation of IL-10

**Fig 3:**
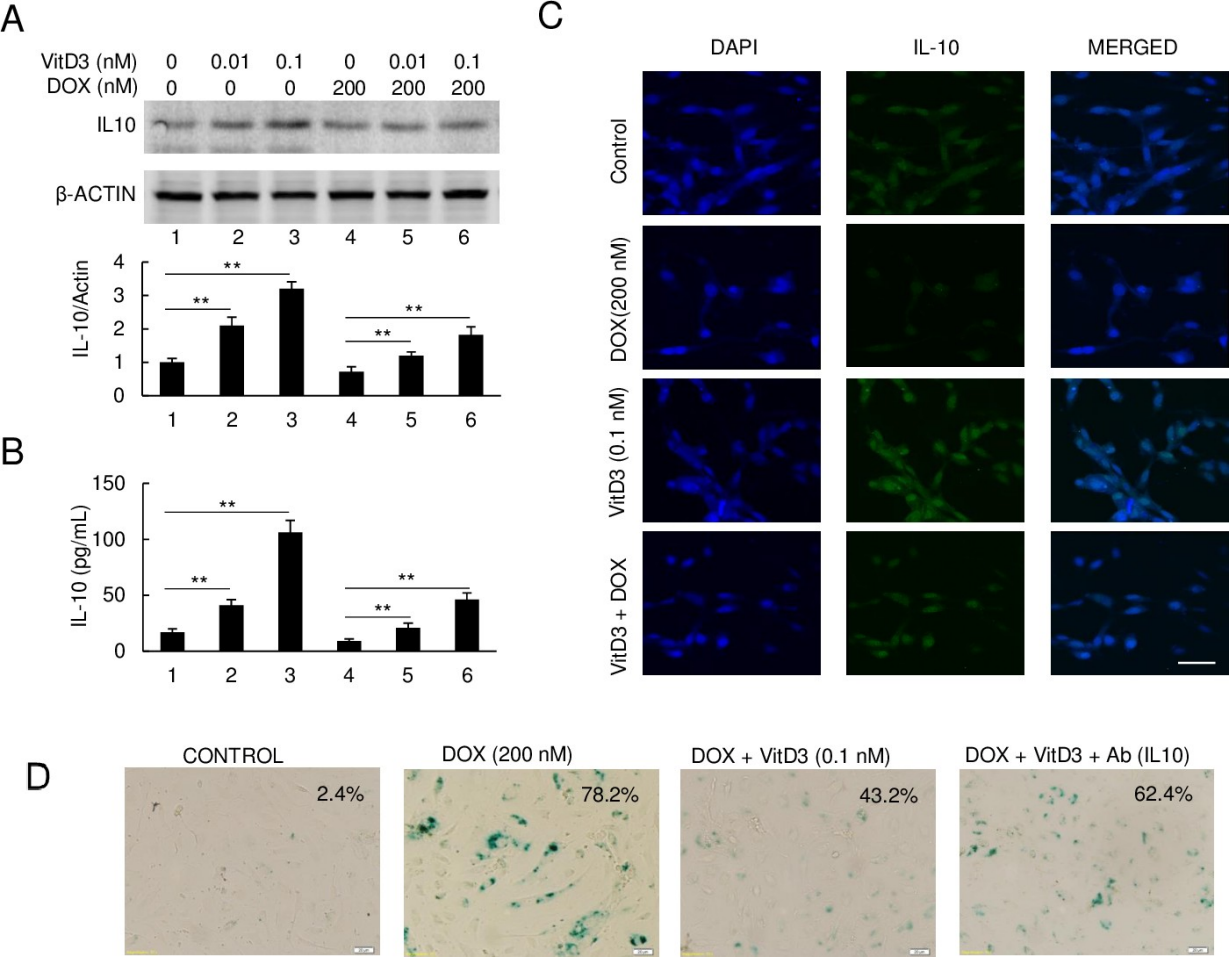
VitD3 upregulates IL-10 expression to attenuate DOX-induced senescence. (A) Immunoblot of IL-10 expression after DOX and VitD3 cotreatment. The bottom panel is the quantified density of protein levels (**p < 0.01, n = 3). (B) ELISA measurement of IL-10 levels in the culture media (**p < 0.01, n = 3). (C) Fluorescence staining of IL-10 on HAEC cells under VitD3 and DOX cotreatment. IL-10 was stained with FITC-labeled and DAPI was performed for nucleus staining. Scar bar, 10 μm. (D) IL-10 antibody attenuated VitD3 protection effect.

We next measured the IL-10 level changes after DOX and/or VitD3 treatment. Fig 3A shows the results of immunoblot assay and corresponding densitometric data respectively. IL-10 levels increased in HAECs by about 2 and 3-fold with 0.01 nM and 0.1 nM of VitD3 treatment respectively. Furthermore, cotreatment with 0.1 nM of VitD3 increased IL-10 levels around 1.8-fold compared to DOX-only treated HAECs. To further validate and confirm our findings, IL-10 ELISA was performed to quantify the levels of IL-10 in culture media. Shown in Fig 3B, IL-10 levels in culture media of HAECs that were untreated and treated with 0.01 nM and 0.1 nM of VitD3 were 17 pg/ mL, 41 pg/mL, and 106 pg/mL for the untreated HAECs, 0.01 nM VitD3, and 0.1 nM VitD3 respectively. The levels of IL-10 in DOX treated HAEC culture media were significantly reduced at 9 pg/mL. In cotreatment groups, levels were 21 pg/mL in 0.01 nM of VitD3 + DOX and 46 pg/mL in 0.1 nM of VitD3 + DOX. Similar results were obtained with immunofluorescent staining for IL-10 (Fig 3C). Compared with the control, a strong fluoresce signal was observed in 0.1 nM of VitD3 treated cells, while DOX strongly suppressed the IL-10 fluorescence signal. The intermediate fluorescence signal was also observed in the DOX + VitD3 group of HAECs, which is much stronger than DOX-only treated HAECs (Fig 3C).

To examine whether IL-10 is an essential factor involved in VitD3 protection against DOX-induced senescence in HAECs, IL-10 specific antibody was added to the culture media. We observed the addition of IL-10 antibody strongly attenuating the protection effect of VitdD3 on DOX-induced senescence of HAECs (Fig 3D). These data indicate VitD3 has a protective effect on DOX-induced senescence of HAECs primarily via the upregulation of IL-10.

### The expression of IL10 induced by VitD3 is through AMPKα/SIRT1/FOXO3a signaling

**Fig 4:**
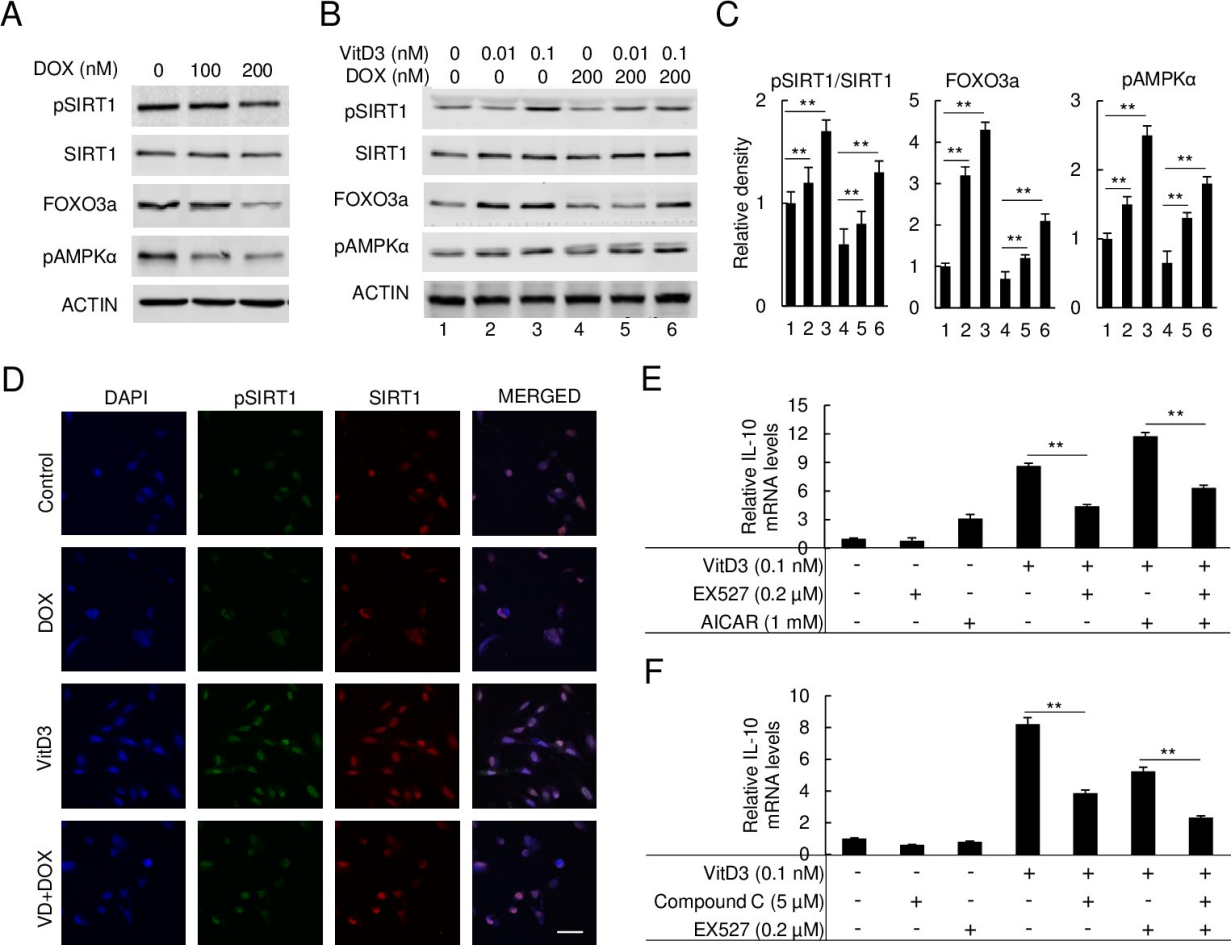
VitD3 directly activates pAMPKα/SIRT1/FOXO3a signal to regulate IL-10 expression. (A) Immunoblot of FOXO3a, pAMPKα, SIRT1, and pSIRT1 expression levels under DOX treatment. (B and C) Immunoblot of FOXO3a, pAMPKα, SIRT1, and pSIRT1 expression levels under DOX and VitD3 co-treatment and the relative density of protein levels was quantified (**p < 0.01, n = 3). (D) The immunofluorescence staining of pSIRT1 and SIRT1 on HAECs. pSIRT1 was stained as FITC and SIRT1 as TRITC. DAPI was performed as a nucleus staining as control. Scar bar, 10 μm. (E and F) qPCR analyzed the relative *IL-10* mRNA expression under VitD3, SIRT1 inhibitor, EX527, AMPKα activator, AICAR, and inhibitor, compound C (**p < 0.01, n = 3).

To investigate the molecular cascade by which VitD3 modulates IL-10-mediated protective effects, the AMPKα, SIRT1, and FOXO3a signaling pathways were analyzed. Immunoblot of DOX-treated HAECs reveals pSIRT1, FOXO3a, and pAMPKα expression levels were markedly reduced by DOX in a dose-dependent manner (Fig 4A). As described in Fig 4B and 4C, VitD3 strongly enhanced the expression of pSIRT1, FOXO3a, and pAMPKα in the VitD3-only. During cotreatment with VitD3 and DOX, we observed that VitD3 significantly restored the expression of pSIRT1, FOXO3a, and pAMPKα compared to the DOX-only treatment group (Fig 4B and 4C). Fig 4D shows results of immunofluorescent staining, revealing the expression of pSIRT1 was strongly induced by VitD3 and suppressed by DOX, while VitD3 + DOX had partial restoration of pSIRT1 expression. Unexpectedly, SIRT1 expression was also observed to be slightly induced by VitD3 treatment, which was barely affected by the administration of DOX (Fig 4B).

To confirm the role of pSIRT1, FOXO3a, and pAMPKα signaling in VitD3-mediated IL-10 expression, we performed qPCR to quantify the relative mRNA expression levels of *IL-10*. We further examined the mRNA expression levels of *IL-10* after treatments with a SIRT1 specific inhibitor (EX527), an AMPKα activator (AICAR), and an AMPKα inhibitor (compound C). As in Fig 4E and 4F, both 0.1 nM VitD3 and 1 mM AICAR strongly induced *IL-10* mRNA levels independently and more so after cotreatment with both. SIRT1 inhibitor, EX527, significantly suppressed IL-10 mRNA levels and attenuated the increased *IL-10* expression by VitD3-only, and AICAR-only, and VitD3 + AICAR groups. The AMPKα inhibitor, compound C, was tested next to quantitatively assess the role of AMPKα in VitD3 induced *IL-10* expression. Compound C was observed to have a function similar with EX527 in suppressing *IL-10* mRNA levels. Compound C was also observed to strongly suppress *IL-10* mRNA expression when combined with VitD3-only or VitD3 + EX527 groups (Fig 4F). Collectively, this set of data indicates the AMPKα/SIRT1 axis regulates VitD3 mediated IL-10 expression.

### SIRT1/FOXO3a complex directly interacts with the IL-10 promoter region to induce its expression

**Fig 5:**
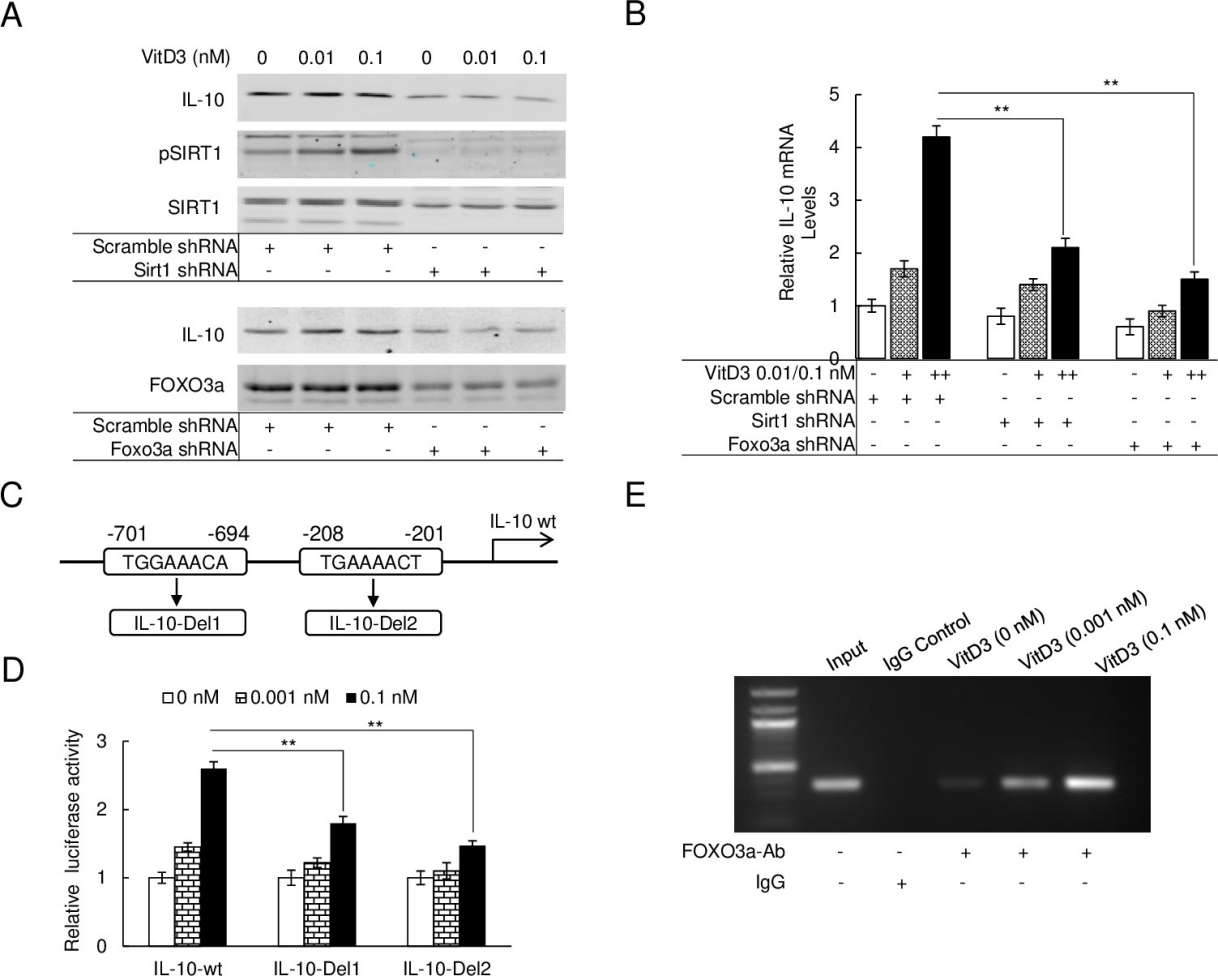
SIRT1/FOXO3a complex directly interacts with the IL-10 promoter region to induce its expression. (A) Immunoblot of IL-10 expression levels treated with VitD3 in SIRT1 knocking down HAECs (upper) or FOXO3a knocking down HAECs (bottom). Scramble shRNA transfected as control. (B) qPCR to quantify the mRNA levels of *IL-10* on the VitD3 treated SIRT1 knocking down HAECs or FOXO3a knocking down HAECs (**p < 0.01, n = 3). (C) Genomic analysis of the promoter region and two traditional FOXO3a binding domains were acquired on -701 to -694 (TGGAAACA) and -208 to -201 (TGAAAACT). Gene modification to delete either of these two fragments and named as IL-10-Del1 or IL-10-Del2. (D) Luciferase reporter assay performed with IL-10-wt, IL-10-Del1 or IL-10-Del2 under VitD3 treatment (**p < 0.01, n = 3). (E) CHIP assay performed with the FOXO3a antibody under VitD3 treatment of HAECs.

To further analyze the SIRT1/FOXO3a signal pathway, SIRT1 or FOXO3a specific shRNA was used to knock-down SIRT1 or FOXO3a expression in HAECs. The scramble-shRNA transfected HAECs were designed as the wild-type control. Immunoblots were performed on both the SIRT1 or FOXO3a knockdown and scramble control HAECs to examine IL-10 expression during VitD3 administration. As seen in Fig 5A, the immunoblot showed a lack of VitD3-mediated increased expression of IL-10 in SIRT1 and FOXO3a knockdown HAECs. In wild-type HAECs, however, IL-10 expression regulated by VitD3 was strongly increased in a dose-dependent manner. Moreover, a parallel decline in expression of pSIRT1 was seen in SIRT1 knockdown HAECs treated with VitD3 (Fig 5A). Using qPCR, we further examined the *IL-10* mRNA expression levels in wild-type and SIRT1 or FOXO3a knockdown HAECs. As shown in Fig 5B, we observed an upregulation of *IL-10* mRNA levels on scramble control HAECs after VitD3 treatment. In contrast, a slight increase of mRNA expression was observed on transgenic SIRT1 and FOXO3a knocked down cells. Taken together, these expression profiles further support that VitD3 regulates IL-10 expression is through the AMPKα/SIRT1/FOXO3a signal pathway.

To determine whether the SIRT1/FOXO3a complex is a transcriptional activator for IL-10, the human *IL-10* genomic sequence was analyzed for potential FOXO3a response elements. Two potential response elements were identified in the promoter region of *IL-10* (Fig 5C) with scores greater than 0.8 from online promoter analysis software (https://jaspardev.genereg.net/). The promoter region of *IL-10* with FOXO3a binding sites was further modulated and subcloned into a firefly-luciferase reporting vector and pGL3-Basic, and named as pGL3-IL-10-wt, pGL3-IL-10-Del1, and pGL3-IL-10-Del2 respectively to quantify the response of SIRT1/FOXO3a after VitD3 treatment (Fig 5C). Fig 5D highlights the induced luciferase signal by 1.45 and 2.6-fold after 0.001 nM and 0.1 nM treatment with VitD3 respectively in the pGL3-IL-10-wt group. In contrast, a significantly reduced luciferase signal was noted in pGL3-IL-10-Del1 and pGL3-IL-10-Del2 groups after VitD3 treatments. To investigate the binding activity of the SIRT1/FOXO3a complex to these two potential elements, chromatin immunoprecipitation (ChIP) assays were performed for IL-10 on the VitD3 responses of treated HAECs. The results of the ChIP assay indicated that the SIRT1/FOXO3a complex directly binds to the promoter region of *IL-10* in VitD3 treated groups (Fig 5E). Collectively, these data suggest VitD3 regulates increases in IL-10 expression through activation of the SIRT1/FOXO3a complex, which acts as a direct transcriptional promotor.

### VitD3 modulates FOXO3a activity through both transcription and nuclear translocation

**Fig 6:**
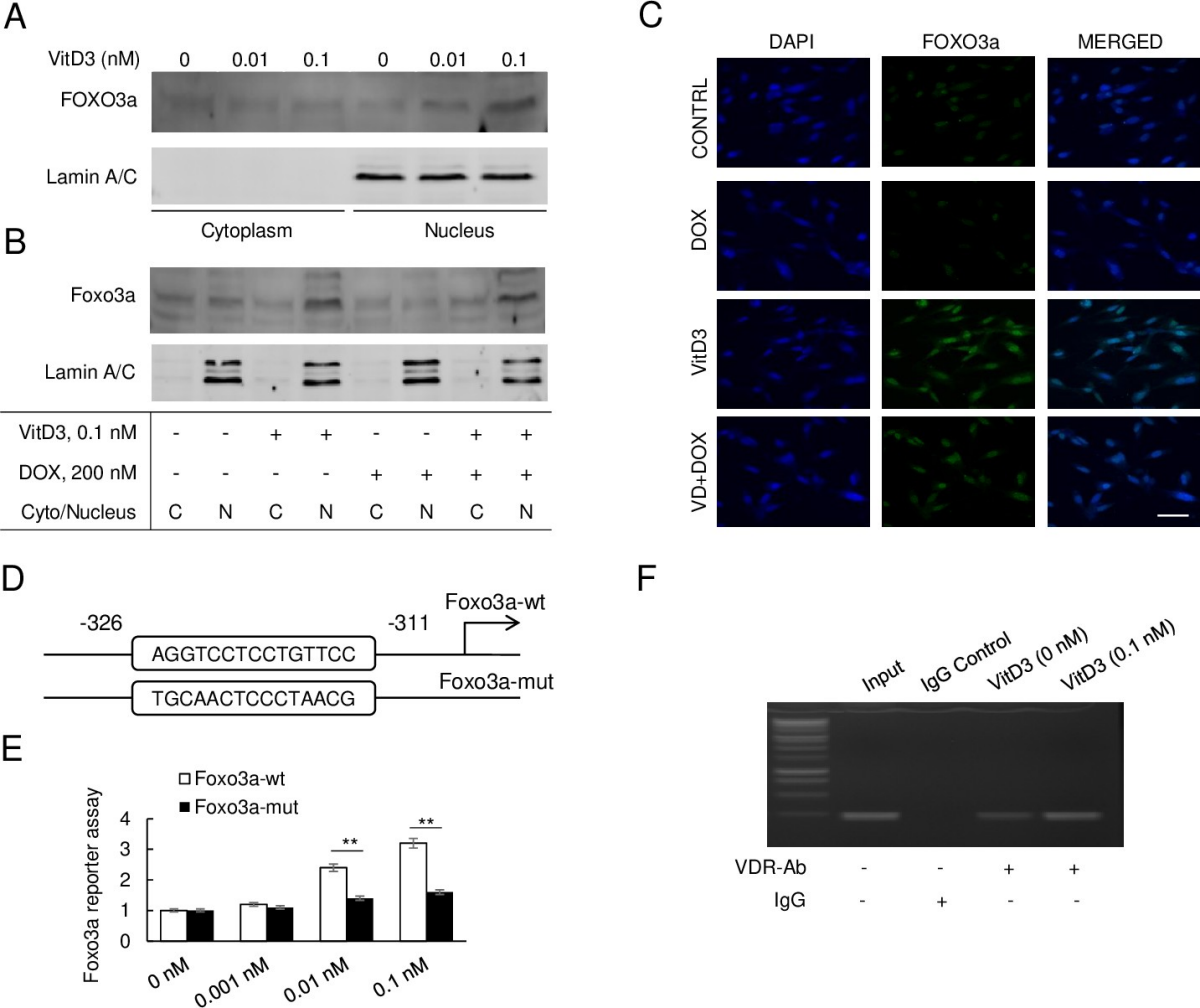
VitD3 modulates FOXO3a activity through both transcription and nuclear translocation. (A) Immunoblot of FOXO3a in cytoplasm and nucleus of VitD3 treated HAECs, Lamin A/C as nucleus control. (B) Immunoblot of FOXO3a in cytoplasm or nucleus of HAECs under VitD3 and/or DOX treatment. (C) Immunofluorescence staining of FOXO3a under VitD3 and DOX treatment. DAPI as nucleus control. Scar bar, 10 μm. (D) Genomic analysis of the promoter region and a traditional VDR binding domain was acquired on -326 to -311 (AGGTCCTCCTGTTCC). Gene modification mutated this fragment and named as Foxo3a-mut. (E) Luciferase reporter assay performed with Foxo3a-wt, or Foxo3a-mut under VitD3 treatment. (F) CHIP assay performed with the VDR antibody under VitD3 treatment of HAECs.

To further understand the VitD3-mediated regulation of FOXO3a activity, the cytoplasmic and nuclear fractions of FOXO3a from VitD3-treated HAECs were collected and an immunoblotting assay was performed with FOXO3a antibody. Immunoblot of FOXO3a from the cytoplasm and the nucleus implied that most of the FOXO3a translocated to the nucleus when treated with VitD3. Moreover, the nuclear translocation of FOXO3a induced by VitD3 was dose-dependent (Fig 6A). FOXO3a nuclear translocation was further analyzed after VitD3 and DOX cotreatment. As Fig 6B shows, VitD3-only treatment strongly amplified FOXO3a nuclear translocation. Conversely, this was significantly neutralized in the DOX-only treatment group and partly restored in the DOX + VitD3 group. The immunofluorescence staining of HAECs with FOXO3a specific antibody further confirmed the above findings with a strong fluorescence signal and partly restored fluorescence signal in VitD3-only group and DOX + VitD3 group respectively. As seen in Fig 6C, the fluorescence signal of the nucleus was much stronger in the VitD3-only group when compared to the DOX-only and DOX + VitD3 groups.

Besides increased nuclear translocation, VitD3 was also observed to upregulate FOXO3a expression. To investigate the mechanism, the genomic DNA sequence of *FOXO3a* was analyzed. We observed a vitamin D receptor (VDR) binding fragment located at the *FOXO3a* promoter region (Fig 6D). A luciferase reporter assay was further performed to investigate the expression of FOXO3a under VitD3 administration. As described in Fig 6E, VitD3 boosted the FOXO3a luciferase signal in a dose-dependent response. After the mutation of the VDR binding domain in the *FOXO3a* promoter region, only a slight increase in the luciferase signal was observed compared to the wild-type control (Fig 6E). To further investigate the interaction of the VDR and *FOXO3a* promoter region, ChIP assay with VDR specific antibody was performed on VitD3 treated HAECs. As compared to the IgG antibody and VitD3-nontreated control, specific VDR binding was observed in the *FOXO3a* genomic area. Moreover, VDR binding to the *FOXO3a* genomic area was more dominant in the VitD3 treated group (Fig 6F). These results indicated VitD3 directly upregulates FOXO3a expression through VitD3-VDR complex binding to its promoter area.

## Discussion

The primary findings of our study are as follows: 1. VitD3 protects against DOX-induced senescence of HAECs primarily via upregulation of IL-10 expression. 2. VitD3 induces IL-10 through fine modulation of AMPKα/SIRT1/FOXO3a signaling pathway, which is strongly inhibited by DOX. 3. VitD3 treatment directly upregulates FOXO3a expression by binding to a vitamin D receptor (VDR) complex which leads to FOXO3a nuclear translocation. 4. SIRT1/FOXO3a acts as a direct transcriptional promotor for IL-10. Based on these findings, we propose a mechanism for VitD3 protection against DOX-induced senescence of HAECs through the increased expression of IL-10 via the AMPKα/SIRT1/FOXO3a signaling pathway.

Senescence is a phenomenon by which cellular proliferation is irreversibly halted [23]. The senescence of vascular tissue promotes vascular dysfunction, accelerates arteriosclerosis, and ultimately enhances the susceptibility and magnitude of DOX-induced cardiotoxicity [24]. DOX is known to induce senescence in endothelial cells [3, 4]. However, the mechanisms of this process and possible interventions are not well-explored. Numerous studies have indicated that IL-10 is a potent vascular cytokine and has been shown to reduce hypertension and inflammation-mediated senescence of the vasculature [25–28]. In this study, we showed DOX decreases IL-10 expression in HAECs, inducing senescence. Thus, strategies to increase IL-10 levels may have therapeutic potential in treating DOX-induced senescence and, therefore, limit overall toxicity on the human cardiovascular system.

There is evidence that VitD3 can promote IL-10 expression in various cell lines and human clinical trials [29, 30]. The mechanisms, however, remain unclear. In this study, we found FOXO3a is an essential factor, regulated by VitD3, for boosting IL-10 levels through both post‑translational modifications (PTMs) and expression levels.

Studies have shown kinases phosphorylate FOXO3a, leading to its transcript inhibition through suppression of nuclear translocation and protein degradation [31]. AMPKα is unique because it leads to FOXO3a activation through phosphorylation of FOXO3a without affecting the cytoplasmic-nuclear shuttling of FOXO3a [32]. This indicates AMPKα influences FOXO3a activity only when it is in the nucleus. Conversely, there are conflicting studies suggesting AMPKα may facilitate FOXO3a nuclear localization [33]. The exact effect of FOXO3a transactivation by AMPKα remains unclear. However, a potential molecular mechanism through which AMPKα regulates FOXO3a is the recruitment of additional partners to the transcriptional complex of FOXO3a to target promoter regions. In this study, we observed that DOX strongly suppress pAMPKα and pSIRT1 levels, which could be counteracted with the administration of VitD3. This suggests that AMPKα and SIRT1 are the major modulators of FOXO3a during VitD3 upregulation of IL-10 expression. Several studies have implied that AMPKα and SIRT1 both regulate each other and share many common target molecules [34–36]. By using specific inhibitors of SIRT1 and AMPKα, and by knocking-down SIRT1 and FOXO3a expression, we believe VitD3-mediated IL-10 expression is through the post-translational activities of FOXO3a by way of the SIRT1 and AMPKα axis. Besides the PTMs by AMPKα/SIRT1 axis, we also observed VitD3 can directly induce FOXO3a expression by binding to its promoter area. Thus, VitD3 mediated IL-10 upregulation and its protective effects on HAECs against DOX-induced chemotoxicity are mediated via AMPKα/SIRT1/FOXO3a signaling pathway.

VitD3 is known to regulate homeostasis in different tissues, including skeletal muscle, vascular smooth muscle, myocardium, and endothelium with a beneficial effect on cardiovascular function [12, 37, 38]. VitD3 is also known to have protective effects against angiotensin II and irradiation-induced oxidative stress and senescence, and VitD3 deficiency is known to accelerate aging, age-related senescence, and inflammation, especially in the nervous and cardiovascular system [39–42]. Conversely, in cancer cells, VitD3 is routinely used with DOX in cancer patients and is known to sensitize breast cancer cells to DOX by potentiating its oxidative toxic potential [16, 18]. Although the protective effects of VitD3 and IL-10, as well as the role of the AMPKα/SIRT1/FOXO3a signaling pathway, have been studied both in vivo and in vitro against oxidative stress, aging, and metabolic syndrome, we have demonstrated the protective effects of VitD3 against DOX-induced senescence in HAECs by upregulation of IL-10. Furthermore, we also unpinned the molecular mechanism by which VitD3 upregulates IL-10. Our findings are impactful in the exploration of newer targets for intervention against cardiovascular complications during DOX administration in cancer patients. Altogether, VitD3 appears to be a safe choice as a potential target to limit DOX-induced toxicity on the cardiovascular system in cancer patients.

In summary, we propose VitD3 as a novel agent to limit DOX-induced senescence of HAECs though upregulation of IL-10 via the AMPKα/SIRT1/FOXO3a pathway. Further studies, especially involving in vivo models, are required to confirm our findings and explore the molecular mechanism in more detail.

## Supporting information

S1 Raw images (PDF)

S1 Table Key resources table. (PDF)

S2 Table Sequences of oligonucleotides used in this study. (PDF)
