# Supplementary figures and images for: Vitamin D3 attenuates doxorubicin-induced senescence of human aortic endothelial cells by upregulation of IL-10 via the pAMPKα/Sirt1/Foxo3a signaling pathway
Source: PLoS One. 2021 Jun 8;16(6):e0252816. doi: 10.1371/journal.pone.0252816 (PMC8186764; doi:10.1371/journal.pone.0252816)

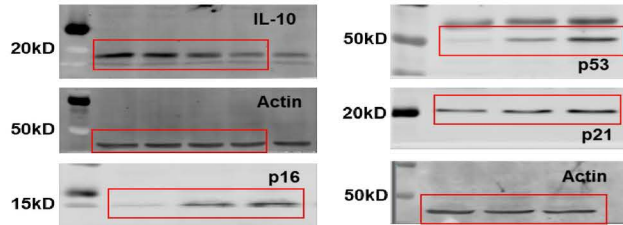

Fig. 1A, 1D

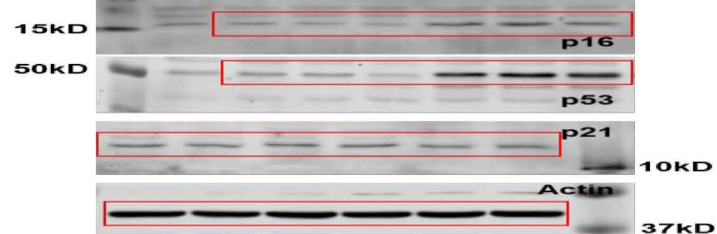

Fig. 2B

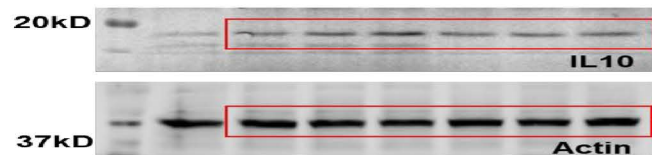

Fig. 3A

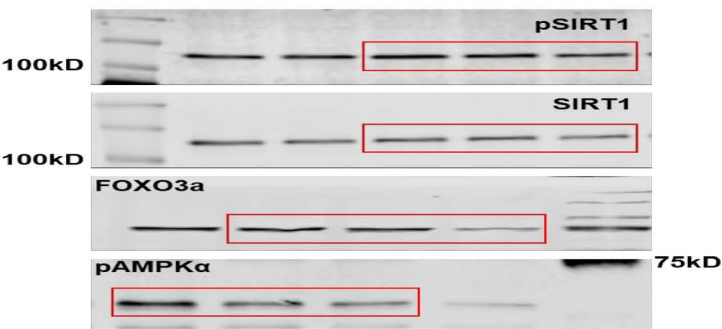

Fig. 4A

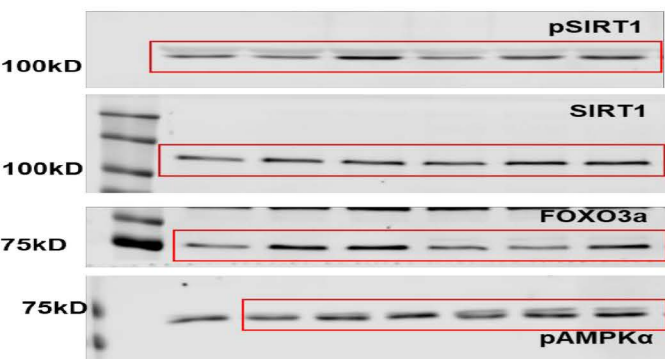

Fig. 4B

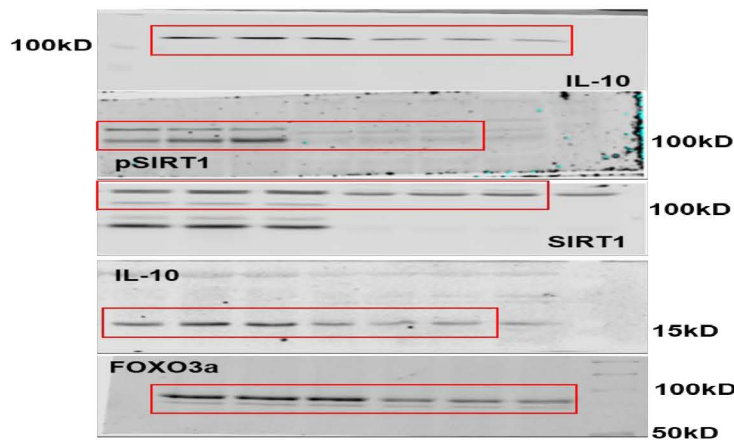

Fig. 5A

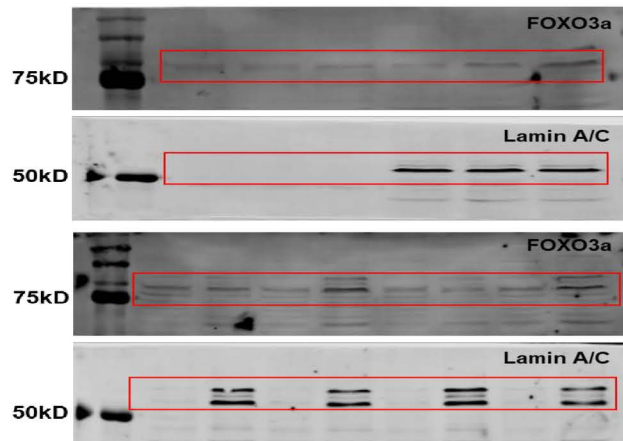

Fig. 6A, 6B

Supplement: S1 Raw images — (PDF) [file pone.0252816.s001.pdf]
