# Supplementary material for: Vitamin D3 attenuates doxorubicin-induced senescence of human aortic endothelial cells by upregulation of IL-10 via the pAMPKα/Sirt1/Foxo3a signaling pathway
Source: PLoS One. 2021 Jun 8;16(6):e0252816. doi: 10.1371/journal.pone.0252816 (PMC8186764; doi:10.1371/journal.pone.0252816)
Supplement: S1 Table — (PDF) [file pone.0252816.s002.pdf]

S1 Table: Key Resources Table

REAGENT OR RESOURCE	SOURCE	IDENTIFIER
**Antibodies**		
anti-IL-10	Novus	MAB9184
anti-p16	Cell Signaling Technology	80722
anti-p21	Cell Signaling Technology	2947, RRID:AB_823586
anti-p53	Cell Signaling Technology	9282, RRID:AB_331476
anti-VDR	Cell Signaling Technology	12550, RRID:AB_2637002
anti-SIRT1	Cell Signaling Technology	8469, RRID:AB_10999470
anti-pSIRT1	Cell Signaling Technology	2314, RRID:AB_561516
anti-FOXO3a	Cell Signaling Technology	2497, RRID:AB_836876
anti-pAMPKα	Cell Signaling Technology	2535, RRID:AB_331250
anti-ß-Actin	Santa Cruz Biotechnology	SC-1616, RRID:AB_630836
anti-Lamin A/C	Cell Signaling Technology	2032, RRID:AB_2136278
anti-human IL-10 mAb 9D7	Mabtech	3430-3N-500
DyLight 488 Horse Anti-Rabbit IgG	Vector Laboratory	DI-1088, RRID:AB_2336403
DyLight 594 Horse Anti-Rabbit IgG	Vector Laboratory	DI-1094, RRID:AB_2336414
DyLight 488 Horse Anti-Mouse IgG	Vector Laboratory	DI-2488, RRID:AB_2307439
DyLight 549 Horse Anti-Mouse IgG	Vector Laboratory	DI-2549, RRID:AB_2336406
IRDye® 800CW Donkey anti-Rabbit IgG	LI-COR Biosciences	925-32213, RRID:AB_2715510
IRDye® 680RD Donkey anti-Mouse IgG	LI-COR Biosciences	925-68072, RRID:AB_2814912
Normal Rat IgG1	Santa Cruz Biotechnology	SC-3882, RRID: AB_737232
**Chemicals, Reagents, and Media**		
DMEM	Fisher	11965092
HECG Media	PromoCell	C-22010
FBS	Fisher	10-437-028
0.05% Trypsin/EDTA	Fisher	25300054
EX527	MilliporeSigma	E7034
Compound C	MilliporeSigma	171260
AICAR	MilliporeSigma	A9978
2% gelatin	MilliporeSigma	G1393
Puromycin	MilliporeSigma	P8833
Doxorubicin hydrochloride	MilliporeSigma	D1515
1α,25-Dihydroxyvitamin D3	MilliporeSigma	D1530
Phusion High-Fidelity DNA Polymerase	NEB	M0530
TRIzol reagent	Invitrogen	15596018
EverGreen qPCR master mix (2x)	Midscience	BEQPCR-LR
BSA	Milliporesigma	A1933
Pierce IP lysis buffer	Fisher	87788
Protease inhibitors	Roche Diagnostics	04693124001
Lipofectamine^{TM} 3000	Invitrogen	L3000015
**Critical Commercial Assays**		

Senescence β-Galactosidase Staining Kit	Cell Signaling Technology	9860
IL-10 ELISA kit	Invitrogen	ENEHIL10
BD PI/RNase Staining Buffer	BD Biosciences	BDB550825
Monarch DNA Gel Purification Kit	NEB	T1020
Cytofix/Cytoperm fixation/permeabilization kit	BD Biosciences	554715
BCA protein assay kit	Fisher	23224
First-strand cDNA synthesis system	Bio-Rad	170-8841
Dual-Glo™ Luciferase Assay System	Promega	PR-E2920
Q5 site-directed mutagenesis kit	NEB	E0554
**DNA, Vectors, and shRNA virus particles**		
pGL3-basic vector	Promega	E1751
pRL-TK vector	Promega	E2241
pLKO.1-SIRT1 shRNA particles	MilliporeSigma	TRCN0000229630
pLKO.1-FOXO3a shRNA particles	MilliporeSigma	TRCN0000010335
pLKO.1-puro Non-Target shRNA Control	MilliporeSigma	SHC016V
pEZX-IL10 (NM_000572)	Genecopoeia	HPRM30536
pEZX-FOXO3a (NM_001455)	Genecopoeia	HPRM43701
**Cell**		
Primary human aortic endothelial cells	Cell Application Inc	304k-05a
**Software and Algorithms**		
FlowJo	https://flowjo.com	
Jaspardev	https://jaspardev.genereg.net/	
NEBaseChanger	https://nebasechanger.neb.com/	
Image Studio	https://www.licor.com/bio/image-studio/	
