# Supplementary material for: Vitamin D3 attenuates doxorubicin-induced senescence of human aortic endothelial cells by upregulation of IL-10 via the pAMPKα/Sirt1/Foxo3a signaling pathway
Source: PLoS One. 2021 Jun 8;16(6):e0252816. doi: 10.1371/journal.pone.0252816 (PMC8186764; doi:10.1371/journal.pone.0252816)
Supplement: S2 Table — (PDF) [file pone.0252816.s003.pdf]

S2 Table: Sequences of oligonucleotides used in this study, Related to the Methods

**PRIMER FOR qPCR**		
**NAME**	**SEQUENCE (5' to 3')**	**GenBank Accession**
IL10-F	TCAAGGCGCATGTGAACTCC	NM_000572
IL10-R	GATGTCAAACTCACTCATGGCT	
FOXO3a-F	TCACGCACCAATTCTAACGC	NM_001455
FOXO3a-R	CACGGCTTGCTTACTGAAGG	
TP53-F	GAGGTTGGCTCTGACTGTACC	NM_001126118
TP53-R	TCCGTCCCAGTAGATTACCAC	
**PRIMERS FOR CHIP ASSAY**		
**NAME**	**SEQUENCE (5' to 3')**	
CHIPFOXO3-F1	AGTATTTCCACACCGCGATAA	
CHIPFOXO3-F1	CGAGAGTCTCAACGACAACAA	
CHIPFOXO3-F2	CTGGTGTCGCGTTCTAACA	
CHIPFOXO3-F2	CCCTCCCGAGAGTCTCAA	
CHIPIL10-F1	CACAGGGAGGATGAGTGATTTG	
CHIPIL10-R1	CTGGATAGGAGGTCCCTTACTT	
CHIPIL10-F2	CTTTAGACTCCAGCCACAGAAG	
CHIPIL10-R2	ATGTGTTCCAGGCTCCTTTAC	
**PRIMERS FOR FOXO3a PROMOTER CONSTRUCTION**		
**NAME**	**SEQUENCE (5' to 3')**	
FOXO3-KpnI-F	CTGGGTACCCAGCGGGGACAGC	
FOXO3-SmaI-R	CGCCCGGGGAAGCACCAAAGAA	
FOXO3a-mut-F	CCTAACGTGGGAGGCGGGCGCGGCA	
FOXO3a-mut-R	GAGTTGCAGAAGACGTGCGGCCAGCGAACCTGG	
**PRIMERS FOR IL-10 PROMOTER CONSTRUCTION**		
**NAME**	**SEQUENCE (5' to 3')**	
IL-10-Del1-F	GTGCCTGAGAATCCTAATGAAATC	
IL-10-Del1-R	CCTCTTCAGCTGTCCCCC	
IL-10-Del2-F	AAGTTTATTAGAGAGGTTAGAG	
IL-10-Del2-R	ATTTTTGCATCGTAAGCAAAAATG	
IL-10-KpnI-F	CTGGTACCTAGGTCAGTGTTCCTCCCA	
IL-10-XhoI-R	CACTCGAGCAAGCCCCTGATGTGTAGA	
